# A 22-pJ/spike 73-Mspikes/s 130k-compartment neural array transceiver with conductance-based synaptic and membrane dynamics

**DOI:** 10.3389/fnins.2023.1198306

**Published:** 2023-08-28

**Authors:** Jongkil Park, Sohmyung Ha, Theodore Yu, Emre Neftci, Gert Cauwenberghs

**Affiliations:** ^1^Center for Neuromorphic Engineering, Korea Institute of Science and Technology (KIST), Seoul, Republic of Korea; ^2^Institute for Neural Computation, University of California, San Diego, La Jolla, CA, United States; ^3^Department of Electrical and Computer Engineering, Jacobs School of Engineering, University of California, San Diego, La Jolla, CA, United States; ^4^Department of Bioengineering, Jacobs School of Engineering, University of California, San Diego, La Jolla, CA, United States; ^5^Division of Engineering, New York University Abu Dhabi, Abu Dhabi, United Arab Emirates; ^6^Peter Grünberg Institute, Forschungszentrum Jülich, RWTH, Aachen, Germany

**Keywords:** neuromorphic cognitive computing, integrate-and-fire array transceiver (IFAT), address event representation (AER), conductance-based synapse, dendritic computation, log-domain translinear circuits, asynchronous pipelining, rectified linear unit (ReLU)

## Abstract

Neuromorphic cognitive computing offers a bio-inspired means to approach the natural intelligence of biological neural systems in silicon integrated circuits. Typically, such circuits either reproduce biophysical neuronal dynamics in great detail as tools for computational neuroscience, or abstract away the biology by simplifying the functional forms of neural computation in large-scale systems for machine intelligence with high integration density and energy efficiency. Here we report a hybrid which offers biophysical realism in the emulation of multi-compartmental neuronal network dynamics at very large scale with high implementation efficiency, and yet with high flexibility in configuring the functional form and the network topology. The integrate-and-fire array transceiver (IFAT) chip emulates the continuous-time analog membrane dynamics of 65 k two-compartment neurons with conductance-based synapses. Fired action potentials are registered as address-event encoded output spikes, while the four types of synapses coupling to each neuron are activated by address-event decoded input spikes for fully reconfigurable synaptic connectivity, facilitating virtual wiring as implemented by routing address-event spikes externally through synaptic routing table. Peak conductance strength of synapse activation specified by the address-event input spans three decades of dynamic range, digitally controlled by pulse width and amplitude modulation (PWAM) of the drive voltage activating the log-domain linear synapse circuit. Two nested levels of micro-pipelining in the IFAT architecture improve both throughput and efficiency of synaptic input. This two-tier micro-pipelining results in a measured sustained peak throughput of 73 Mspikes/s and overall chip-level energy efficiency of 22 pJ/spike. Non-uniformity in digitally encoded synapse strength due to analog mismatch is mitigated through single-point digital offset calibration. Combined with the flexibly layered and recurrent synaptic connectivity provided by hierarchical address-event routing of registered spike events through external memory, the IFAT lends itself to efficient large-scale emulation of general biophysical spiking neural networks, as well as rate-based mapping of rectified linear unit (ReLU) neural activations.

## 1. Introduction

Neuromorphic systems implementing spiking neural networks are promising research platforms for investigating and emulating the computational abilities of the brain (Mead, 1990; Indiveri et al., 2011; Thakur et al., 2018). The compactness and low-power consumption of neuromorphic circuits make them highly suited for robotic and mobile applications emulating the dynamics of complex brain circuits in real-world environments (Badoni et al., 2006; Indiveri et al., 2006; Silver et al., 2007; Schemmel et al., 2010; Merolla et al., 2011; Ramakrishnan et al., 2012; Sharp et al., 2012; Imam and Cleland, 2020). Such complex real-life tasks require large-scale neuromorphic systems, and there are various approaches for their implementation. They range from implementations using microprocessor cores integrated with specialized network-on-chip routers (Furber et al., 2012; Sharp et al., 2012; Painkras et al., 2013), fully digital implementations with quasi-asynchronous elements to maintain synchrony (Merolla et al., 2011, 2014; Imam et al., 2012; Akopyan et al., 2015), SRAM-based implementations for programmable precision of neural and synaptic dynamics and connectivity in a core and supporting local learning rules (Davies et al., 2018; Detorakis et al., 2018; Frenkel et al., 2019), implementations using amplifier-based neuron circuits with wafer-scale integration and connectivity (Schemmel et al., 2010; Millner et al., 2011; Schmitt et al., 2017), analog quadratic integrate-and-fire neurons sharing synapses, axons, and dendrites with neighboring neurons implementing a diffusive neural network as layered in the cortex (Lin et al., 2006; Benjamin et al., 2014; Neckar et al., 2019), and subthreshold CMOS analog neurons with digitally controlled conductance-based synapses (Yu et al., 2012b; Park et al., 2014). Despite the success of large-scale implementations, the required synaptic density of the scale of the brain with neuronal dynamic representations at low power consumption remains a challenge.

All these neuromorphic systems are built from basic neural computation units, that is neurons and synapses, which are also the basic computational elements in the biological brain. A neuron processes incoming information and transmits its outputs using an electrical signal represented by an action potential to other neurons via synapses. A basic principle for the emulation of neural and synaptic dynamics is the integration of synaptic currents into the membrane potential and generation of action potentials. There are various models for emulating these principles (Destexhe et al., 1998). Some neuron models emulate neural dynamics in more biologically plausible ways, ranging from a model of ion channel kinetics with hundreds of differential equations and parameters (Hodgkin and Huxley, 1952) to models of simplified conductance-based differential equations for computational efficiency (Izhikevich, 2003; Mihalas and Niebur, 2009). However, the hardware complexity for the implementation of these neuron models limits the large-scale integration of neurons in a silicon die. Conversely, the leaky integrate-and-fire neuron model is a popular choice for large-scale implementation because of its relative simplicity and ability to emulate many dynamic features of biological neurons (Brette and Gerstner, 2005). The integrate-and-fire neuron models the synaptic current integration and the generation of the action potential. A neuron generates an action potential when the membrane potential exceeds a certain threshold voltage. This basic principle can be implemented using a comparator and an integrator; thus, this simplicity makes it suitable for large-scale implementation in a silicon die.

When a presynaptic neuron generates a spike, it releases neurotransmitters to the synapses connected to postsynaptic neurons. In the biological brain, a neuron is connected to 10,000 neurons on average. Achieving hard-wired synaptic connections to the level of the biological brain is highly challenging in neuromorphic hardware. This challenge can be addressed using the asynchronous address event representation (AER) protocol in neuromorphic systems. AER facilitates spike event communication between arrayed neurons using address events, each of which represents a target neuron address with synaptic parameters (Sivilotti, 1991; Lazzaro et al., 1993; Mahowald, 1994; Deiss et al., 1999; Boahen, 2000). When a neuron fires in an array, the spike is encoded as an address event representing the address of the neuron in the array. The event is translated to synaptic events through a synaptic routing table implemented in random access memory (RAM) or read-only memory (ROM), and these synaptic events are sent to postsynaptic neurons. In each postsynaptic neuron, an incoming synaptic event accumulates the membrane potential of the postsynaptic neuron.

An integrate-and-fire array transceiver (IFAT) is proposed and developed as a promising system platform for large-scale power-efficient neuromorphic processing. In our previous studies, integrate-and-fire neurons were arranged in a 2 k-neuron core (with 2,048 neurons), and each neuron used a simple analog-switched capacitor architecture to model membrane dynamics, resulting in a discrete-time version of synaptic current integration (Goldberg et al., 2001; Vogelstein et al., 2007). This demonstrated the ability to emulate a model of attractor dynamics and neural activity in the rat hippocampus. For a more compact form of synapses while further extending the linearity of the synaptic dynamics in continuous time, a single-transistor realization of a conductance-based synapse emulating the log-domain encoding of first-order linear dynamics of synaptic conductance was presented (Yu and Cauwenberghs, 2010). In addition, large-scale integration incorporating a hierarchical AER architecture has been realized (Yu et al., 2012a; Park et al., 2017). For address event routing, a synchronous AER circuit was placed for each 2 k-neuron core. In this scheme, an event holds the AER circuit until the event is delivered, thus resulting in a limited input event throughput. In this study, the AER protocol is implemented fully asynchronously, implying that there is no synchronized system clock. The AER protocol is only activated by address events with a “handshaking” protocol. When a sender and receiver are ready to communicate, they send and receive a request and acknowledge signal to deliver an event. This event-driven activation reduces the dynamic power consumption significantly (Martin and Nystrom, 2006), achieving sub-nanojoule energy efficiency for an asynchronous microcontroller (Martin et al., 2003), and it is also applied to neuromorphic systems for energy-efficient address event communication (Vogelstein et al., 2007; Merolla et al., 2011, 2014; Millner et al., 2011; Benjamin et al., 2014; Davies et al., 2018).

In this paper, we present a 65k-neuron IFAT as a computational building block for large-scale neuromorphic systems. An IFAT neuron comprises two conductively coupled compartments, each with two single-transistor conductance-based synapses. The compact form of single-transistor conductance-based synapses enables the dense integration of 65,536 neurons in a single chip. The IFAT neuron is suitable for continuous-time dynamical emulation of biologically realistic neuronal networks. We demonstrated the proof-of-principles with examples such as multi-compartmental neuronal computation and boundary detection with orientation tuning curves. The synaptic connectivity and event communication in the IFAT rely entirely on the proposed fully asynchronous AER circuits, resulting in low-power consumption owing to its event-driven operation. To maximize the parallelism of the input event streams, an additional pipeline stage was added per row in the 2 k-neuron cores. This two-tier micro-pipeline scheme designed using the asynchronous design principle results in a sustained peak throughput of 73 Mspikes/s at 22 pJ/spike power efficiency.

This paper extends a previous preliminary report Park et al. (2014) which showed the characterizations of a single neuron to the complete characterizations of the entire array of neurons. Additionally, this paper presents the calibration process and mapping of a rate-based neural network onto the architecture with an example of a boundary detection application. The remainder of the paper is organized as follows. In Section 2, we describe the circuit implementation and theoretical motivation behind the implementation. Section 3 presents the measurement results. We show the analysis of a single neuron response and the variability of the response across 2,048 neurons in one core. In addition, we demonstrate a potential application, that is image boundary detection, using IFAT neurons. Section 4 summarizes the related and prior works in a table and discusses potential extensions of the IFAT chip with emerging non-volatile memory devices. Finally, Section 5 concludes the contributions of the IFAT chip.

## 2. Implementation details

### 2.1. Two-compartment integrate-and-fire neuron model

**Figure 1:**
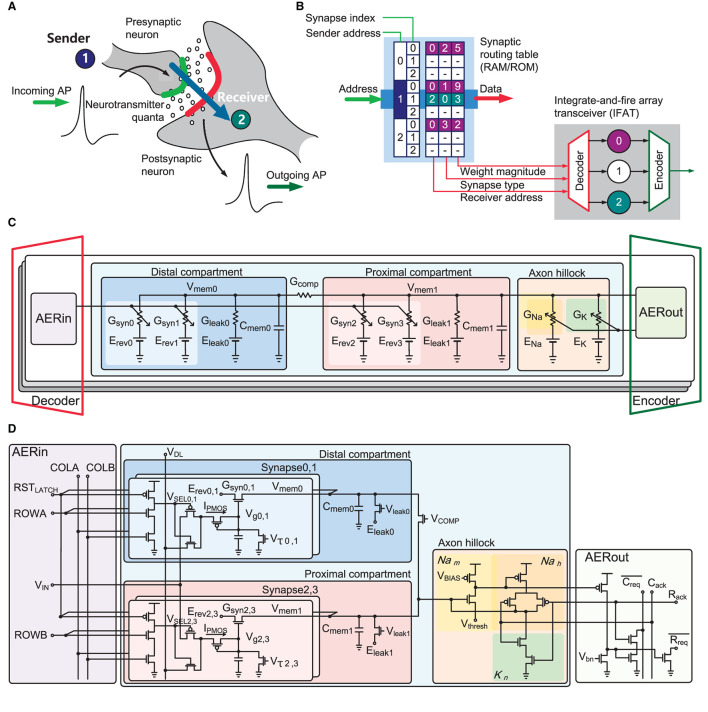
**(A)** Biological neural systems illustrating neural synaptic transmission. Incoming action potential induces that a presynaptic neuron releases neurotransmitters to synapses stimulating a postsynaptic neuron. **(B)** Emulation of the biological neural systems in electronics. Dynamic reconfigurable synaptic connectivity across IFAT arrays using virtual synaptic connections represented in neural spike events through a RAM/ROM synaptic routing table. **(C)** Block diagram of two-compartmental leaky integrated-and-fire neuron model with conductance-based synapses. **(D)** Block diagram and schematic of two-compartment conductance-based leaky integrate-and-fire neuron circuit with AER interface circuits. The proximal and distal compartments, each comprising a conductively leaky membrane with two single-transistor conductance-based synapse circuits, are conductively coupled. A three-transistor dynamic latch holds *V*_*SEL*_ to active low to select one synapse in the selected neuron while a pulse width modulated synaptic input at voltage *V*_*IN*_ activates the synapse. An axon hillock circuit generates action potential and registers output events resetting the membrane potential of proximal compartment *V*_*mem*1_.

The proposed IFAT chip emulates the detailed biological dynamics of neurons and synapses in integrated circuits. Figure 1A illustrates the neural synaptic transmission between neurons. When a presynaptic neuron generates an action potential, it releases neurotransmitters to the synapses, which integrate charges on the membrane of the postsynaptic neuron. When the membrane potential exceeds the firing threshold, the postsynaptic neuron generates an action potential. This neural activation and synaptic communication were emulated in the IFAT chip with a representation of connectivity information in address events, as shown in Figure 1B. Based on such address events using a synaptic routing table, which can be implemented with external memory, such as RAM or ROM, dynamically reconfigurable synaptic connectivity is supported across the IFAT chips in hierarchical address-event routing (HiAER-IFAT) architecture (Park et al., 2017). When a presynaptic neural spike is revieved, synaptic connection information between the presynaptic neuron and its connected postsynaptic neurons is read out from a synaptic routing table, and these address events are routed to the postsynaptic neurons with other synaptic information, which is encoded in the address events, such as synapse type and synaptic weight.

In the IFAT chip, each neuron is implemented using a two-compartment leaky integrate-and-fire neuron model, as shown in Figure 1C. In the neuron model, there are two compartments, called “distal” and “proximal,” each with a membrane capacitor, leak conductance. Each compartment also contained two synapse circuits, which are configured as excitatory or inhibitory synapses by programmable reversal potentials. The synaptic weight modulates the synaptic conductance, defining the amount of current injected into a membrane capacitor in a compartment. Each compartment capacitor is conductively coupled using configurable conductance. When the proximal membrane potential exceeds the threshold voltage, the axon hillock circuit triggers an action potential, similar to the biological system. The dynamics of a two-compartment leaky integrate-and-fire neuron are formulated as follows:


(1) $$\begin{array}{l} C_{mem1}\frac{dV_{mem1}}{dt}=I_{fb}+\underset{j=2,3}{\sum }G_{syn,j}\left(E_{rev,j}-V_{mem1}\right)\\ \;+G_{leak1}\left(E_{leak1}-V_{mem1}\right)\\ \;+G_{comp}\left(V_{mem0}-V_{mem1}\right) \end{array}$$

(2) $$\begin{array}{l} C_{mem0}\frac{dV_{mem0}}{dt}=\underset{j=0,1}{\sum }G_{syn,j}\left(E_{rev,j}-V_{mem0}\right)\\ \;+G_{leak0}\left(E_{leak0}-V_{mem0}\right)\\ \;+G_{comp}\left(V_{mem1}-V_{mem0}\right) \end{array}$$

where *C*_*mem*0_ and *C*_*mem*1_ are the distal and proximal membrane capacitances, respectively; *V*_*mem*0_ and *V*_*mem*1_ are the distal and proximal membrane voltages, respectively; *I*_*fb*_ is the nonlinear positive feedback current due to the spiking mechanism; *G*_*syn*_ is the synapse conductance; *E*_*rev*_ is the reversal potential; *G*_*leak*_ is the leak conductance; *E*_*leak*_ is the leak potential; *G*_*comp*_ is the inter-compartment conductance.

The input and output of a neuron are encoded as address events. A decoder routes an incoming address event to a destination postsynaptic neuron using the information on the synapse type and synaptic weight. Subsequently, an input AER circuit (AERin) stimulates the synapse in the destination neuron with a synaptic weight. On the output side, when an axon hillock circuit registers an event, the output AER circuit (AERout) raises the request signal. An encoder takes the request signal and converts it into an address event, indicating the address of the neuron in the arrayed neurons.

Figure 1D shows a transistor level schematic of the implementation of the two-compartment conductance-based integrate-and-fire neuron. Two conductance-based synapse circuits are tied to a compartment with programmable reversal potentials *E*_*rev*_ defining the synapse type and synaptic time constants controlled by *V*_τ_. In the AERin circuit, an incoming event selects one of the four synapses using pairwise complement signals: rowa, rowb, and cola, colb. Each compartment integrates currents from the synaptic conductance and discharges to continuously leak conductance. In addition, the coupling conductance, which is controlled by the *V*_*COMP*_, couples the electrical charges between the proximal and distal compartments. When the proximal membrane potential exceeds the threshold voltage *V*_*thresh*_, a self-timed axon hillock circuit (Vogelstein et al., 2007) generates an action potential and registers a neural spike event on the AERout circuit to the output AER bus while resetting the membrane potential.

### 2.2. Overall architecture

**Figure 2:**
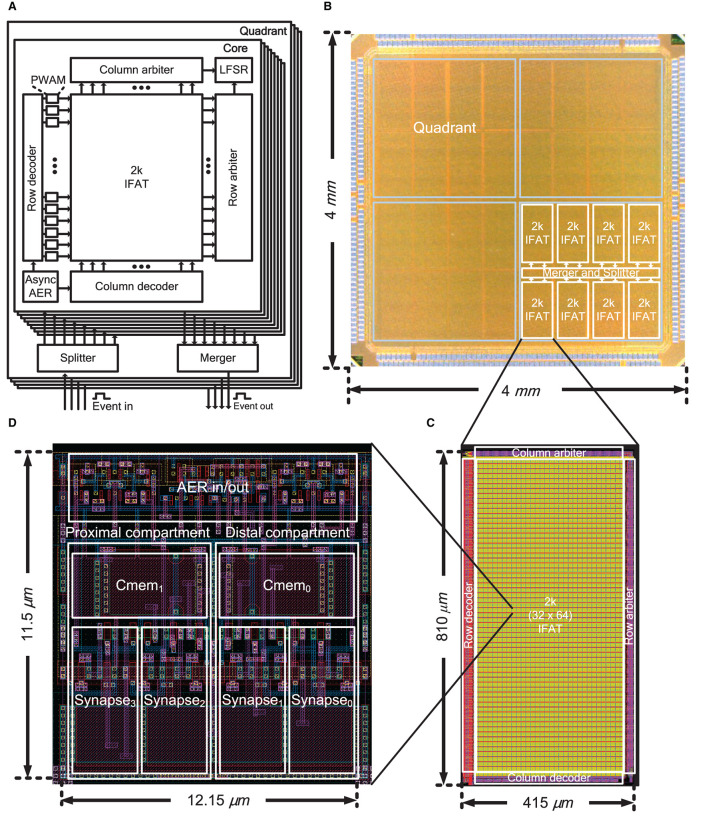
**(A)** Block diagram of the IFAT chip including identical four quadrants each with eight 2 k-neuron IFAT cores, an asynchronous splitter, and an asynchronous merger for event communication. **(B)** Chip micrograph of the IFAT chip. One quadrant, each comprising eight 2 k-neuron IFAT cores and asynchronous AER merger and splitter, is indicated. **(C)** 2 k-neuron IFAT core and **(D)** two-compartment integrate-and-fire neuron cell layout.

Figure 2A shows the overall architecture of the IFAT chip, which is equipped with 65 k integrate-and-fire neurons in a single chip. The 65 k neurons are divided into four independent and identical quadrants, each of which contains eight 2 k-neuron IFAT cores. Each quadrant has independent input and output ports for address event communication. Asynchronous splitters and mergers are placed at the center of each quadrant to control the address event streams from and to the eight 2 k-neuron IFAT cores. Each 2 k-neuron IFAT core comprises 2 ktwo-compartment leaky integrate-and-fire neurons and periphery circuits, such as row and column decoders, pulse width and amplitude modulation (PWAM) circuits, asynchronous AER communication circuit, linear feedback shift register (LFSR), and row and column arbiters. The input and output AER buses are implemented by fully asynchronous communication circuits using a four-phase dual-rail encoding communication protocol. An address event is encoded in the address of the neuron location in the quadrant of the IFAT chip. A previous synchronous pulse-width modulation circuit (Yu and Cauwenberghs, 2010), which incurs a long waiting time between consecutive events, is improved by an additional pipeline stage, row-wise PWAM circuits, which improves the throughput to the 2 k-neuron IFAT core, while the additional amplitude modulation extends the dynamic range of synaptic strength exponentially.

Figure 2B shows a micrograph of the 4 × 4 mm^2^ IFAT chip, which was fabricated using a 90-nm CMOS process. The chip has 436 staggered I/O pads and is packaged in a 35 × 35 mm^2^ Fine Ball Grid Array (FBGA) package. The layouts of the 2 k-neuron IFAT core and neuron cell are shown in Figures 2C, D, respectively. A 2 k-neuron IFAT core occupies 415 × 810 μ*m*^2^ and a two-compartment neuron occupies 12.15 × 11.5 μ*m*^2^.

### 2.3. Conductance-based synapse

**Figure 3:**
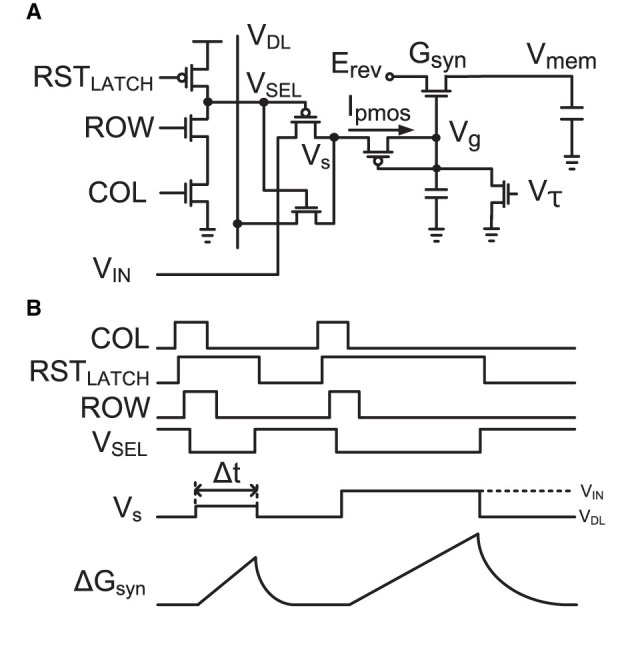
**(A)** Implementation of a single-transistor log-domain conductance-based synapse (Yu and Cauwenberghs, 2010) and a three-transistor dynamic latch. **(B)** Timing diagram of the synapse and dynamic latch operation with two events. When the three-transistor dynamic latch is selected by a row and col, it holds *V*_*SEL*_ to active low for selection of one synapse in the neuron while the pulse width (Δ*t*) modulated input with the amplitude modulated voltage (*V*_*s*_) at *V*_*IN*_, which defines the update of synapse conductance (Δ*G*_*syn*_) according to Equation (6), drives the activated synapse.

Figure 3A shows the single-transistor implementation of a conductance-based synapse (Yu and Cauwenberghs, 2010) incorporating a three-transistor dynamic latch, and Figure 3B shows the timing diagram for its operation. An incoming event drives col and row and sets RST_LATCH_ high, holding *V*_*SEL*_ to active low to select one active synapse in a neuron selected by col and row. Its pMOS diode-connected input is then driven by the source voltage *V*_*s*_. It increases the gate voltage of the synapse *V*_*g*_, increasing the synaptic conductance of *G*_*syn*_ in the log-domain while implementing a linear dynamical synapse with a time constant controlled by *V*_τ_ (Yu et al., 2012a). After a pulse width Δ*t*, *V*_*s*_ returns to *V*_*DL*_, RST_LATCH_ is activated to release *V*_*SEL*_ passive high, and the synapse is ready to receive the next synaptic input event.

The single-transistor conductance-based synapse was conducted in the subthreshold operating regime of the MOS transistor. As explained above, synaptic input events change the conductance of synapse transistors. The synaptic conductance modification in the log domain is formulated from the drain current of the nMOS transistor operating in the subthreshold regime as follows:


(3) $$\begin{array}{l} I_{d}=I_{0}e^{\frac{\kappa V_{g}}{V_{T}}}\left(e^{\frac{-V_{s}}{V_{T}}}-e^{\frac{-V_{d}}{V_{T}}}\right) \end{array}$$

where *I*_0_ is the dark current of the transistor, *V*_*g*_ is the gate voltage, *V*_*d*_ is the drain voltage, *V*_*s*_ is the source voltage, κ is the back gate parameter, and *V*_*T*_ is the thermal voltage. This equation can be transformed to “log-domain” or “pseudo-voltage domain,” with the definition of a pseudo-voltage and pseudo-conductance (Fragnière et al., 1997).


(4) $$\begin{array}{l} I_{d}=G_{syn}\left(E_{rev}-V_{mem}\right) \end{array}$$

where the pseudo-parameters of conductance $G_{syn}=\frac{I_{0}}{V_{T}}e^{\frac{\kappa V_{g}}{V_{T}}}$, reversal potential $E_{rev}=-V_{T}e^{\left(-\frac{V_{d}}{V_{T}}\right)}$, and membrane potential $V_{mem}=-V_{T}e^{\left(-\frac{Vs}{V_{T}}\right)}$.

From the pseudo-parameters of conductance, we can derive the synaptic conductance update with respect to time.


(5) $$\begin{array}{ll} \frac{d}{dt}G_{syn}=\frac{I_{n}}{V_{T}}\frac{d}{dt}e^{\frac{\kappa V_{g}}{V_{T}}}\\ \; & =I_{n}\frac{\kappa }{V_{T}^{2}}e^{\frac{\kappa V_{g}}{V_{T}}}\left(\frac{d}{dt}V_{g}\right)\\ \;=I_{n}\frac{\kappa }{V_{T}^{2}}e^{\frac{\kappa V_{g}}{V_{T}}}\frac{I_{pmos}}{C_{syn}} \end{array}$$

where the back-gate coefficient κ is the same for nMOS and pMOS, *I*_*n*_ and *I*_*p*_ are the subthreshold pre-exponential current factors of nMOS and pMOS, respectively, $I_{pmos}=I_{p}e^{\frac{V_{s}}{V_{T}}}e^{-\frac{\kappa V_{g}}{V_{T}}}$, and *C*_*syn*_ is the synapse capacitor.

The synaptic strength is encoded in pulse width Δ*t* and amplitude *V*_*S*_ modulation, and the resulting step in synaptic conductance Δ*G*_*syn*_ is approximately given by:


(6) $$\begin{array}{l} \Delta G_{syn}=\frac{\kappa I_{n}I_{p}}{V_{T}^{2}C_{syn}}e^{\frac{V_{s}}{V_{T}}}\Delta t\propto \left(1+\frac{W}{16}\right)2^{A} \end{array}$$

where:

- *W* is the relative pulse width of the stimulus, which is the mantissa of the given synaptic strength, in integer units [0, 15], and four least significant bits (LSBs) of eight-bit synaptic strength.
- *A* is the pulse amplitude in the log-domain, which is the exponent of the given synaptic strength in integer units [0, 15], and four most significant bits (MSBs) of eight-bit synaptic strength.

### 2.4. Asynchronous interface with four-phase dual-rail encoding

The AER circuits in the IFAT chip operate in a fully asynchronous way. Asynchronous circuits do not have a master clock for system synchronization. Instead, a “handshaking” protocol is used for reliable data communication between the sender and receiver. Handshaking protocols are implemented with two signals: request and acknowledge. A request signal indicates the sender's readiness to send a data packet. In response to the request signal, the receiver sends an acknowledgment signal back to the sender if available. The sender then sends a data packet. This is an event-driven process. Among various handshaking protocols (Martin and Nystrom, 2006), the IFAT chip uses a four-phase dual-rail encoding protocol for more reliable asynchronous handshaking communication. “Four-phase” means that the whole process of request and acknowledge handshaking comprises four signal-transition phases. “Dual-rail” means that two complementary bit-lines are used to represent one-bit information.

**Figure 4:**
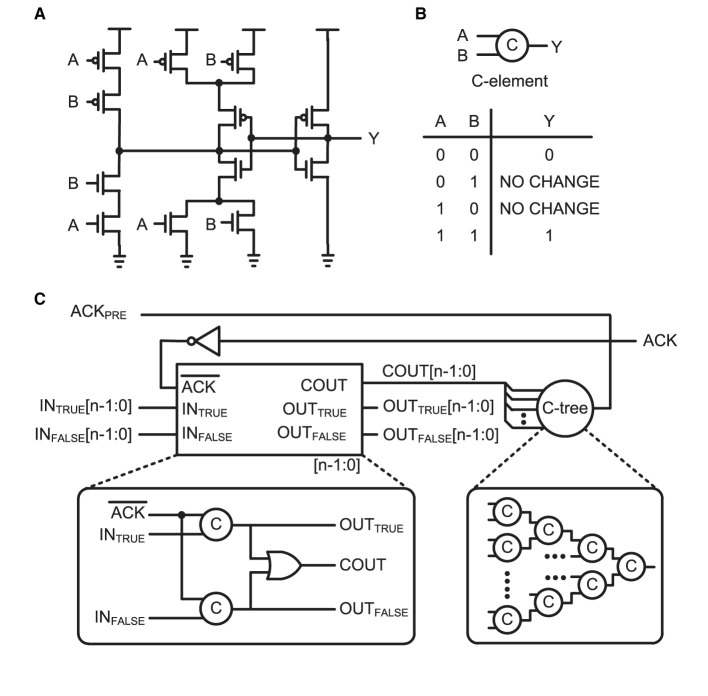
**(A)** Circuit implementation, **(B)** schematic symbol, and truth table of the C-element, which is also called a Muller circuit. **(C)** Schematic of n-bit asynchronous pipeline stage. A one-bit latch with C-elements in dual-rail encoding is shown in the bottom-left box. When the ack is active low, the current stage can latch an input bit. A completion tree (C-tree), which is a tree of C-elements, determines the completion of latched data lines and enables active high to the previous state for the acknowledge signal, ACK_PRE_. The current stage holds the latched data until the next stage acknowledges, via the active high ACK signal.

A basic building block for the protocol is a C-element circuit (Muller circuit; Muller and Bartky, 1957). The circuit implementation, schematic symbol, and truth table of the C-element are presented in Figures 4A, B, respectively. It accepts inputs when the inputs are the same; otherwise, it holds its output value until it receives the same value for both inputs. Such an operation is required for delay-insensitive operations in asynchronous design. Figure 4C shows a schematic of the n-bit asynchronous pipeline stage for the four-phase dual-rail encoding protocol. This pipeline stage holds its data until one of the next pipeline stages is ready to collect the data. It is a function similar to a register in the synchronous design principle. The four-phase dual-rail handshaking protocol does not have an explicit request signal, but it is embedded in the dual-rail. Each bit of the data is encoded in two complementary lines: true and false. The true bit represents the actual value of the data and the false bit is complimentary. If true and false indicate different values, a valid value is loaded into the dual-rail properly, as in true. However, if both are the same, the bit lines are transitioning. The completion tree, the C-tree block shown in Figure 4C, validates that all the bit lines are properly latched. Upon validation, the output of the C-tree is used as an acknowledge signal, ACK_PRE_, to the previous pipeline stage. The properly lathed dual-rail-encoded output bits are considered as a request signal to the next pipeline stage.

### 2.5. Asynchronous splitter and merger

Owing to the limited number of I/O pads on the chip, the input and output buses need to be shared by eight 2 k-neuron IFAT cores in a quadrant. The input bus is designed to communicate 24-bit input synaptic address events. Each event comprised a three-bit destination core address, an 11-bit neuron address in the destination core, a two-bit synapse type, and eight-bit synapse strength. Asynchronous splitters are implemented to locate an input synaptic address event to a destination core. The asynchronous splitter has a binary tree structure of cascaded asynchronous pipeline stages. There are three stages from the input IOs to the destination 2 k-neuron core. At each stage, the MSB of the input synaptic address events is decoded as a request signal to the next pipeline stage.

**Figure 5:**
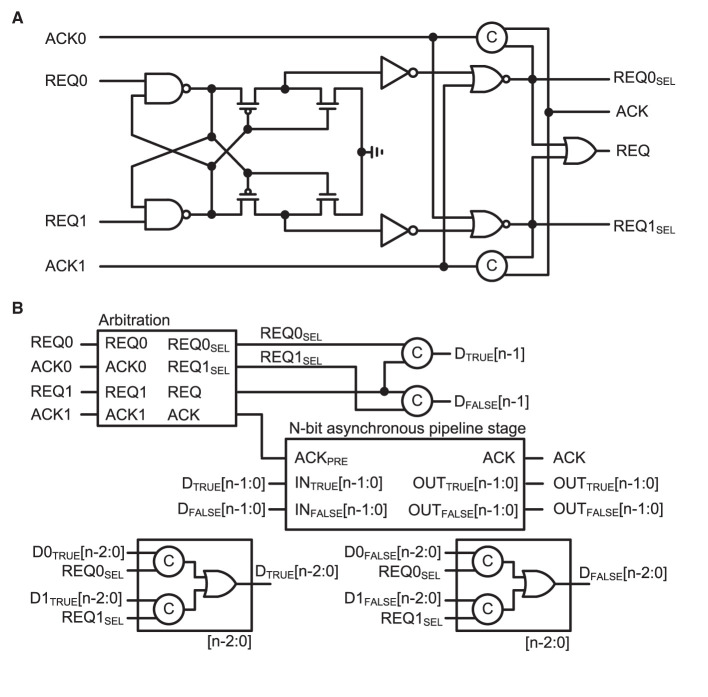
**(A)** Schematic of the arbitration circuit comprising two cross-coupled NAND gates. Two request signals, REQ0 and REQ1, compete to activate one of two cross-coupled NAND-gate paths. The selected request signal enables a path to deliver an acknowledge signal (ACK) to the selected previous stage. **(B)** Block diagram of the asynchronous merger circuit comprising an arbitration circuit and n-bit asynchronous pipeline stage (shown in Figure 4C). N-1 bits are transferred from the selected previous stage, and the selected request signal (REQ0_SEL_ or REQ0_SEL_) is added to the transferred data as the MSB to indicate the source of the data.

On the shared output bus side, an asynchronous merger is designed to multiplex address events that are generated simultaneously from multiple IFAT neuron cores. The asynchronous merger comprised an arbiter and asynchronous pipeline stage. Figure 5 shows the schematics of Figure 5A the arbiter and Figure 5B asynchronous merger circuit. The arbiter circuit receives request signals REQ0 and REQ1 from two paths in the previous stage. Two cross-coupled NAND gates select a path that prioritizes the sending of a request signal to the next signal. The selected request signal, either REQ0_SEL_ or REQ1_SEL_, is encoded in the dual-rail encoding scheme. The dual-rail encoded bit is the MSB of the address event that is selected at the current stage. Additionally, the data from the selected path are properly latched at the asynchronous pipeline stage and acknowledged to be ready for the next event. There are eight 2 k-neuron IFAT cores in each 16 k-neuron quadrant and two paths can be merged using an asynchronous merger. Hence, there are three stages of asynchronous mergers in each quadrant, which are binary-tree structured. When a neuron fires at a 2 k-neuron IFAT core, it is encoded as an 11-bit address event that represents the address of the neuron in the 2 k-neuron IFAT core. One MSB is added to the address event when it passed through each stage, resulting in a 14-bit address event at the output bus of the chip.

### 2.6. Two-tier micro-pipelining scheme

**Figure 6:**
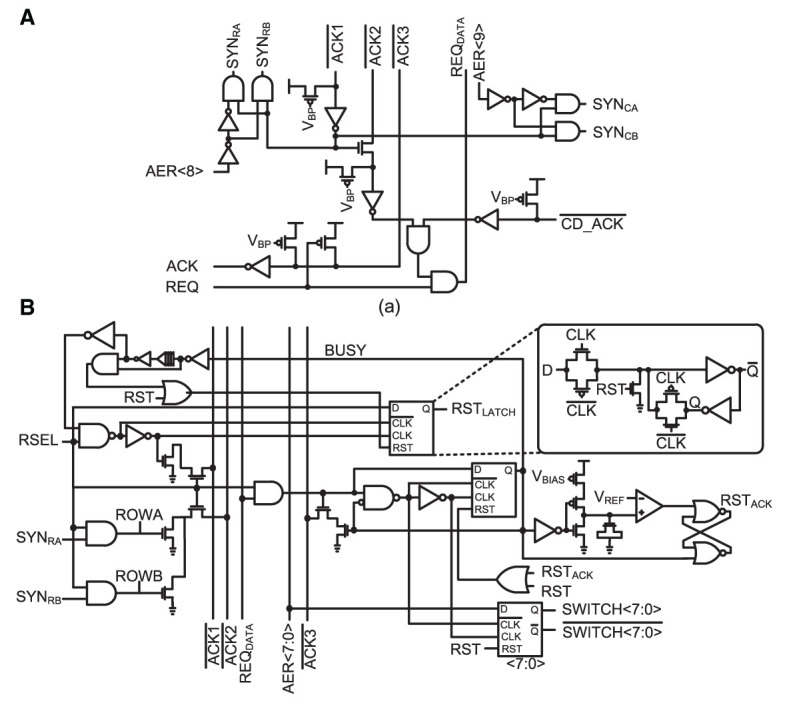
**(A)** Schematic of the input asynchronous AER distribution circuit, which coordinates row-wise PWAM. aer<9:8> in a 21-bit-wide address event determines the synapse type within a selected PWAM circuit. **(B)** Schematic of the PWAM circuit which is in charge of event delivery to a single row. The synaptic strength, which is encoded in aer<7:0>, is delivered with req_DATA_. The switch<7:0> bus latches the synaptic strength for serving an event to a neuron in the row. The four LSBs select the reference voltage of a comparator *V*_*REF*_, which defines the pulse width of the synaptic stimulus. The four MSBs define the amplitude of the stimulus *V*_*s*_.

The communication of each address event at a 2 k-neuron IFAT core is implemented using on-chip asynchronous request (REQ) and acknowledgement (ACK) signals. To increase the throughput of the input events, an input asynchronous AER distribution network on a 2 k-neuron IFAT core is pipelined in two stages with an asynchronous AER communication circuit (shown in Figure 6A) and single-row PWAM circuits (shown in Figure 6B), as shown in Figure 2A. A 2 k-neuron IFAT core receives a 21-bit AER event, which comprises the information of an 11-bit postsynaptic neuron address ([20:10]), a two-bit synapse type ([9:8]), and an eight-bit synapse strength ([7:0]). If a 21-bit AER event is received, the asynchronous AER communication circuit coordinates the AER event to the destination neuron address via column and row decoders and to the synapse type, which is determined by the two-bit synapse type ([9:8]). The asynchronous AER communication circuit then requests a selected PWAM circuit with eight-bit synapse strength. If the PWAM circuit is available, the eight bits for synapse strength are latched onto an eight-bit bus, which selects a comparator reference voltage (*V*_*REF*_) defining the pulse width over the baseline by pulse amplitude (*V*_*IN*_) in the log-domain. If the PWAM circuit is held by a previous address event, the event is not acknowledged and waits until it is serviced.

**Figure 7:**
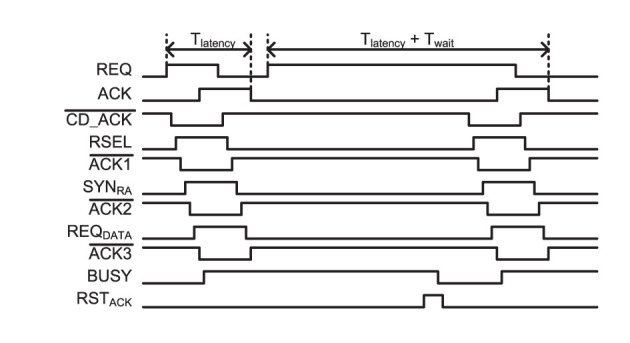
Timing diagram for the input asynchronous AER distribution (Figure 6A) and single-row PWAM (Figure 6B) circuits when two consecutive events address neurons on the same row.

Figure 7 shows a handshaking timing diagram of the two-tier micro-pipelining scheme when two consecutive events address neurons in the same row. It shows asynchronous handshaking timing from a destination neuron address selection via column and row decoders to a selection of synapse types and data packet requests. $\bar{T_{latency}}$ is the latency of handshaking from an asynchronous AER circuit in a 2 k-neuron IFAT core to the destination neuron. If an event is input to the same row as the latest input event, which holds a PWAM circuit, it waits until the event is served to a destination neuron. $\bar{T_{wait}}$ represents the additional latency induced by consecutive input events.

## 3. Measurement results

In this section, we present the experimental results of the system on throughput, system-level energy efficiency, neural activation with respect to input spike strength, and variability due to transistor mismatches across a 2 k-neuron IFAT core. In addition, we present a linear synapse response model with a simple application of orientation tuning curves for boundary detection.

### 3.1. Event throughput

**Figure 8:**
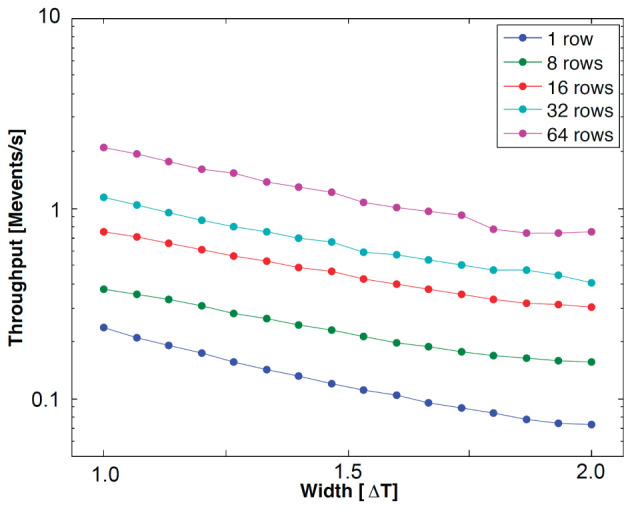
Measured throughput with respect to pulse width representing synaptic strength. The input events address neurons in the same row (1 row) and multiple rows, from 8 to 64.

In the presented architecture, the throughput can be defined as follows:


(7) $$\begin{array}{l} Throughput=\frac{1}{\bar{T_{latency}}+\bar{T_{wait}}} \end{array}$$

where $\bar{T_{latency}}$ is the average event handshaking latency, and $\bar{T_{wait}}$ is the average waiting time in cases where an incoming event addresses a neuron in the same row as the previous event as shown in Figure 7. *T*_*wait*_ is proportional to Δ*t*/*N*_*interleave*_, where Δ*t* is the input pulse width, and *N*_*interleave*_ is the number of interleaved rows. Figure 8 shows the measurement results for event throughput. A spike input stream, which has the maximum pulse width for each input, addressing the 32 neurons in a single row results in 70.6 kevents/s throughput. When the input event stream interleaves multiple rows, the waiting time in a row-wise PWAM circuit is avoided, resulting in higher throughput, as predicted by Equation (7). With this interleaving scheme by the two-tier micro-pipelining stage, we measured 18.2 Mspikes/s per quadrant, and the total throughput of the IFAT chip is thus 73 Mspikes/s.

### 3.2. System-level spike event energy efficiency

**Figure 9:**
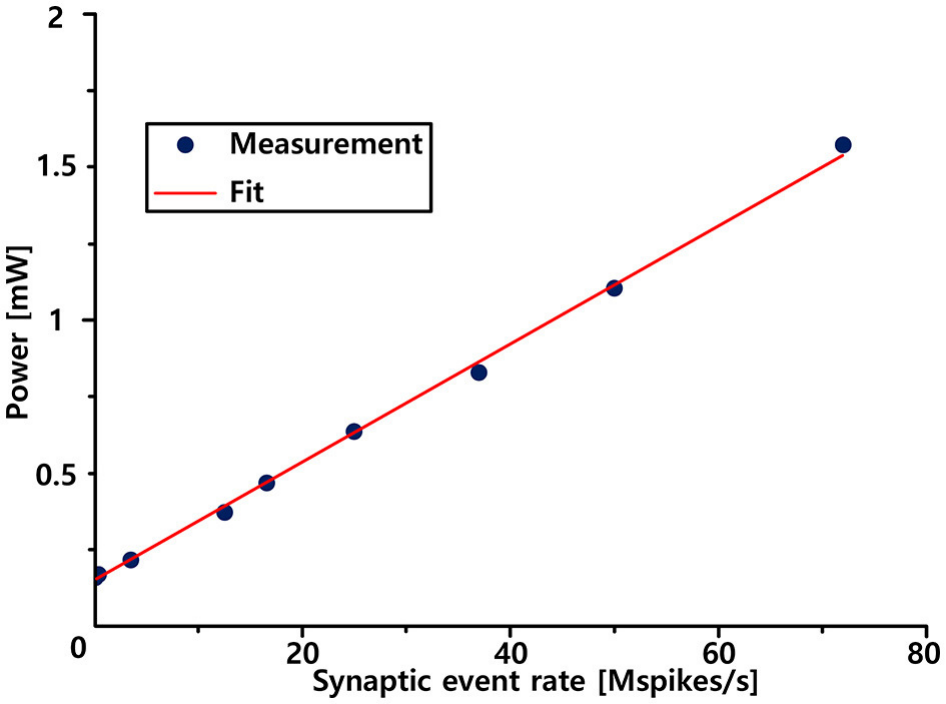
Measured activity-dependent power consumption.

In the brain, each neuron is connected to ~10,000 neurons on average and fires spikes at an average firing rate of 5–10 Hz. Therefore, the power consumption and energy efficiency of biologically inspired neuromorphic systems are primarily determined by synaptic inputs. We then measured the system-level spike event energy efficiency as a function of the synapse input event rate, as shown in Figure 9. This shows that the power consumption increases linearly with the synaptic event input rate. We measured power consumption until the input event rate reached its maximum throughput capability (73 Mevents/s). At the maximum throughput, we measured a current draw of 1.31 mA from a 1.2 V power supply. This resulted in a total power consumption of 1.572 mW. The slope of the graph, which indicates the overall energy efficiency for a spike operation, is measured to be 22 pJ/spike.

### 3.3. Neural activation function

**Figure 10:**
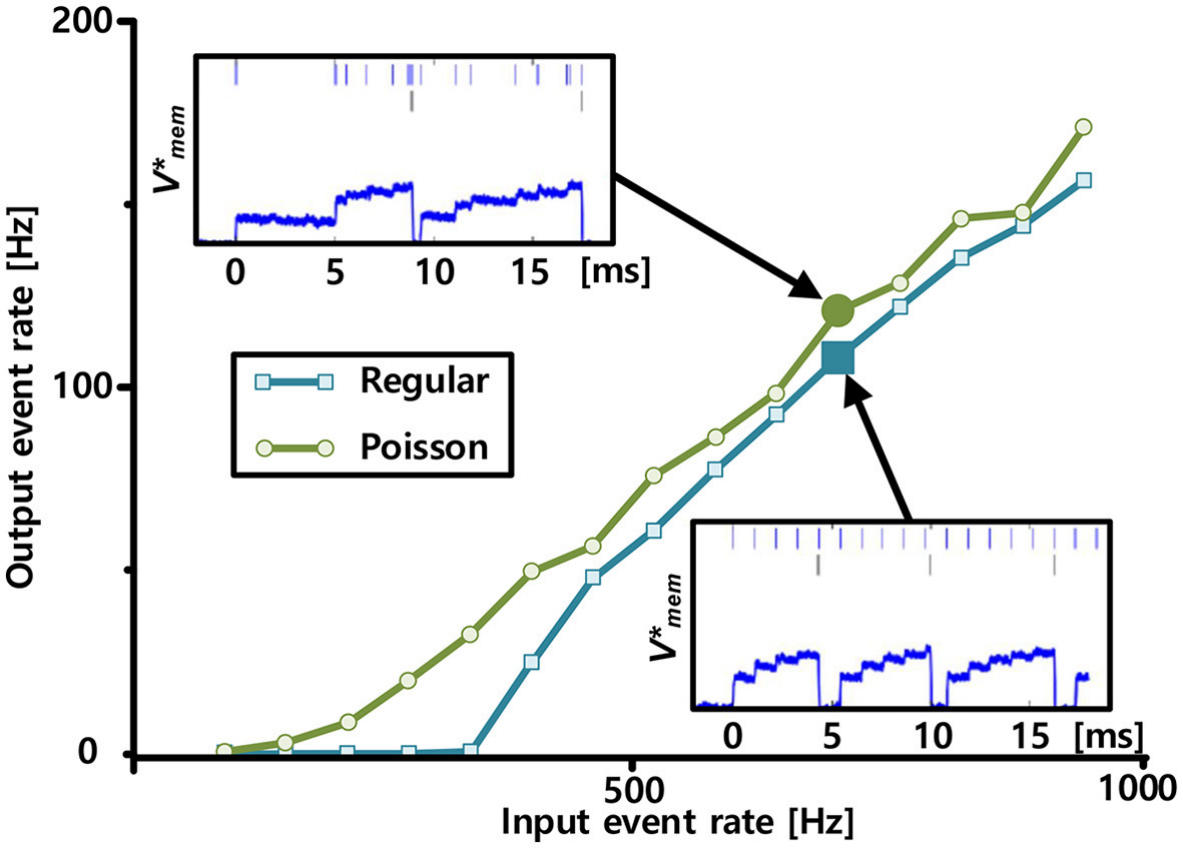
Neural activation functions measured with input spike trains, each comprising Poisson (green) and regular spike trains (blue) with varying input event rates. Measured representative membrane potential, which is shown in the log-domain, from Poisson and regular inputs are plotted on the top left and bottom right insets, respectively. In the insets, input and output spikes are indicated by bars at the top and middle rows, respectively.

Figure 10 shows the neural activation functions, which are defined as the output event rates in response to the input event rates, measured using Poisson and regular spike trains from one representative neuron. The two cases exhibited different activation function shapes. The shape of the function measured using regular input spikes is threshold-linear. This is consistent with the leaky integrate-and-fire neuron model. In the leaky integrate-and-fire neuron model, the threshold originates from the leak conductance of the membrane. In contrast, fluctuations in the Poisson spike trains tend to smooth the activation function, which is expected from studies of noisy integrate-and-fire neuron models (Fusi and Mattia, 1999). In addition, the activation function has a characteristic similar to that of the rectified linear unit model (Nair and Hinton, 2010), which has been widely used in deep neural networks, particularly in convolutional neural networks (CNNs), owing to its faster computation and ability to avoid the vanishing gradient problem.

### 3.4. Multi-compartmental neural computation

A distinguishing feature of the implemented neuron model in the IFAT chip compared to most existing leaky integrate-and-fire neurons is its multi-compartmental neuron implementation. Dendritic computation with proximal and distal compartments in neuroscience exhibits various mechanisms implementing elementary computation units for spatiotemporal information processing (Koch, 1999; London and Häusser, 2005). It has a multiplication-like effect of two time-varying signals in a single neuron resulting in fewer transistors for the implementation, reducing energy and area footprint. Moreover, such neuromorphic dendritic computation shows various applications ranging from configurable multi-layer neural network computation (Ramakrishnan et al., 2013), spatiotemporal input pattern classification by temporal coincidence detection (Wang and Liu, 2013), to efficient learning for event-based sequential data (Yang et al., 2021).

**Figure 11:**
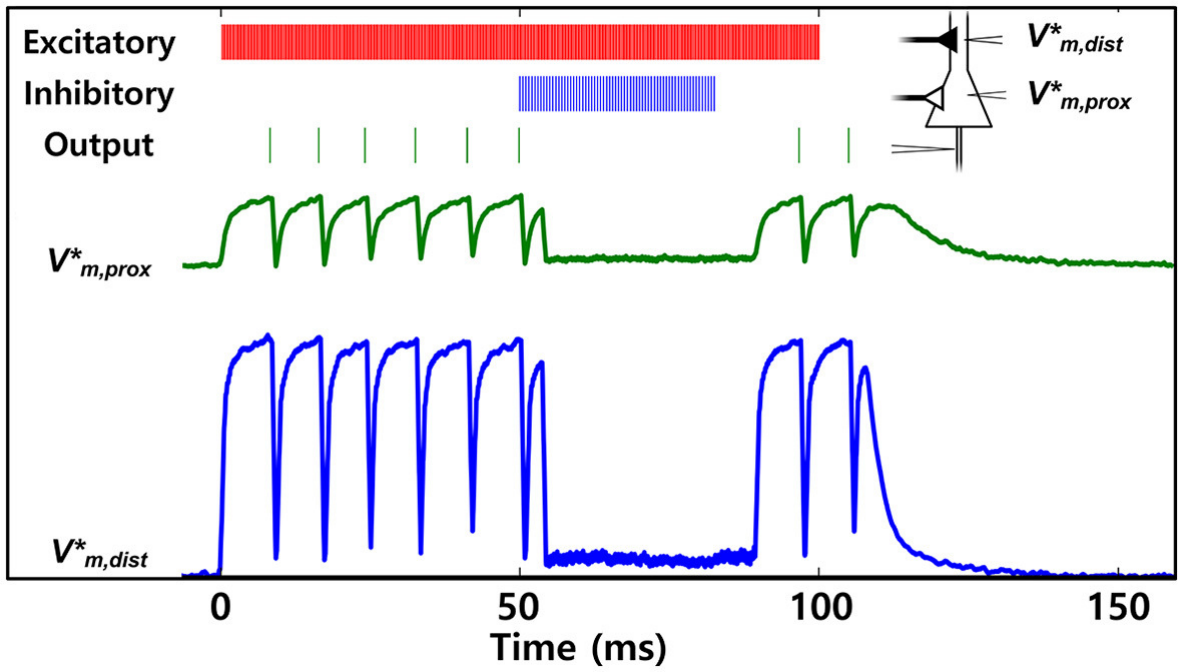
Measured example of shunting inhibition, which blocks the upstream synaptic excitation effect. The distal compartment of the neuron is strongly excited by excitatory synaptic input events, which results in excitatory compartmental inputs coupled through compartmental conductive interactions to the proximal compartment and generation of neuron spike. From 50 to 80 ms, the proximal compartment is inhibited, and then it blocks the upstream synaptic excitation.

The IFAT neuron comprises two compartments: distal and proximal compartments, each with two conductance-based synapses. The compartmental conductances are configurable, implying that the strength of the interaction between the compartments is configurable. Figure 11 shows such interactions as examples of shunting inhibition, which is an important feature of dendritic computation (Nelson, 1994; Mitchell and Silver, 2003; Groschner et al., 2022). Excitatory and inhibitory synaptic inputs, indicated by red and blue bars, respectively, are applied to a neuron, as shown in the schematic. The distal compartment is strongly excited by excitatory synaptic inputs from a regular input spike train. This results in an excitatory compartmental input coupled through the compartment conductance to the proximal compartment of the neuron and the firing of the neuron indicated by green bars in the figure. From 50 to 80 ms, the proximal compartment is inhibited at the reversal potential near rest, which blocks the effect of upstream excitation.

### 3.5. Input-output transfer function of neural response

**Figure 12:**
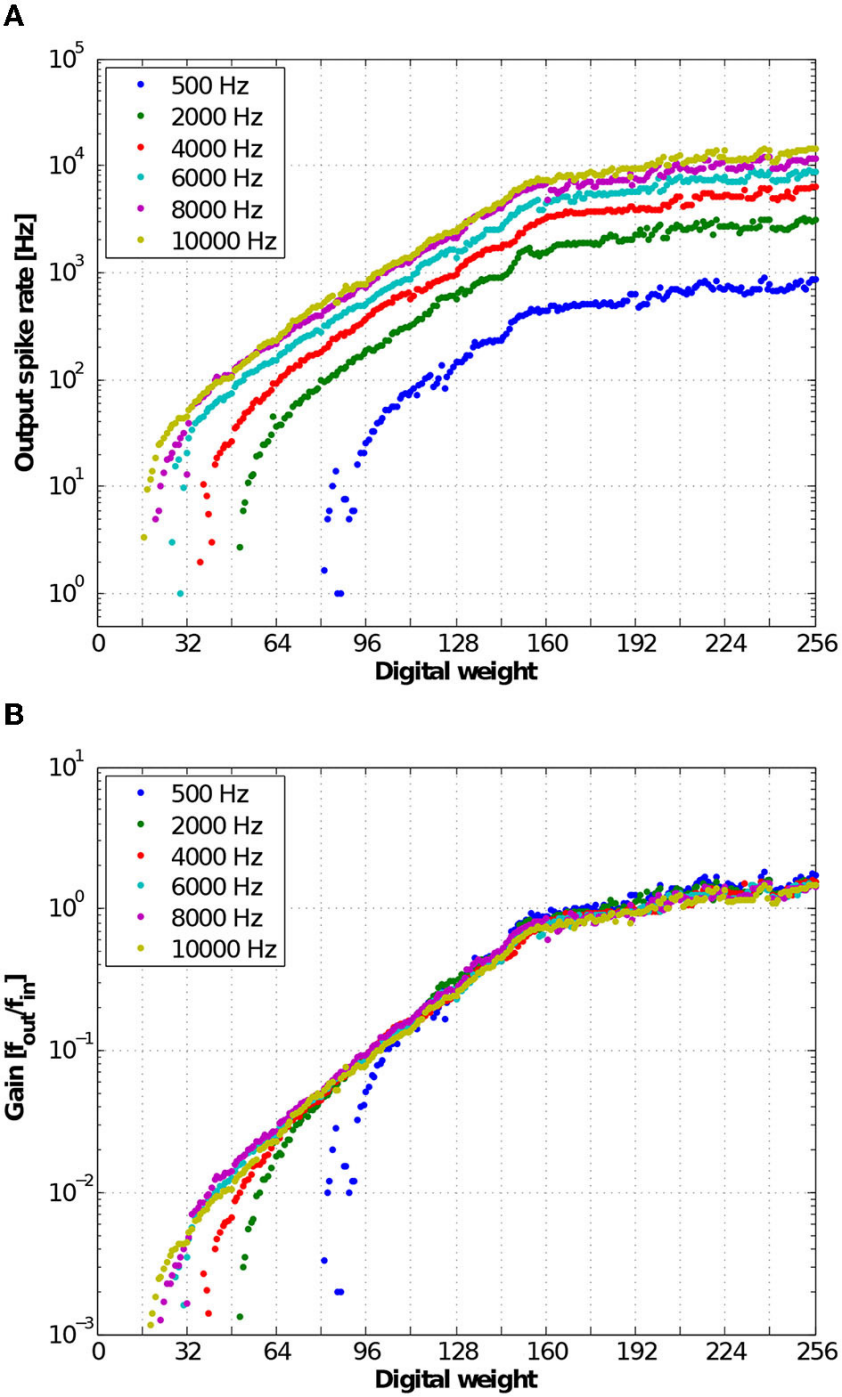
**(A)** Measured input-output transfer function of neural responses. The input spike rate is varied from 500 to 10,000 Hz where the interspike intervals are distributed in the Poisson distribution. **(B)** Measured gain of input-output transfer function of the neuron defined as a ratio of the output and input spike rates.

To characterize the input-output transfer function of the neural response, we measured the output spike rates from one representative neuron over digital weights from 0 to 255 for varying input spike rates from 500 Hz to 10 kHz. To generate Poisson input spike trains, the interspike intervals of the input spike trains were generated using the Poisson process with a constant mean rate. Figure 12A shows the output spike rate of a representative neuron in response to varying digital weights and input spike rates. Figure 12B shows the gain of the neuron, which is the output spike rate normalized by the input spike rate. At a low input spike rate, the membrane potential leaks faster than the synaptic integration, resulting in rare responses at lower digital weights (weak synaptic inputs). At high input strengths, because each input spike produces an output spike, the gain of the input-output transfer function saturates to one.

### 3.6. Neuron mismatch variability

**Figure 13:**
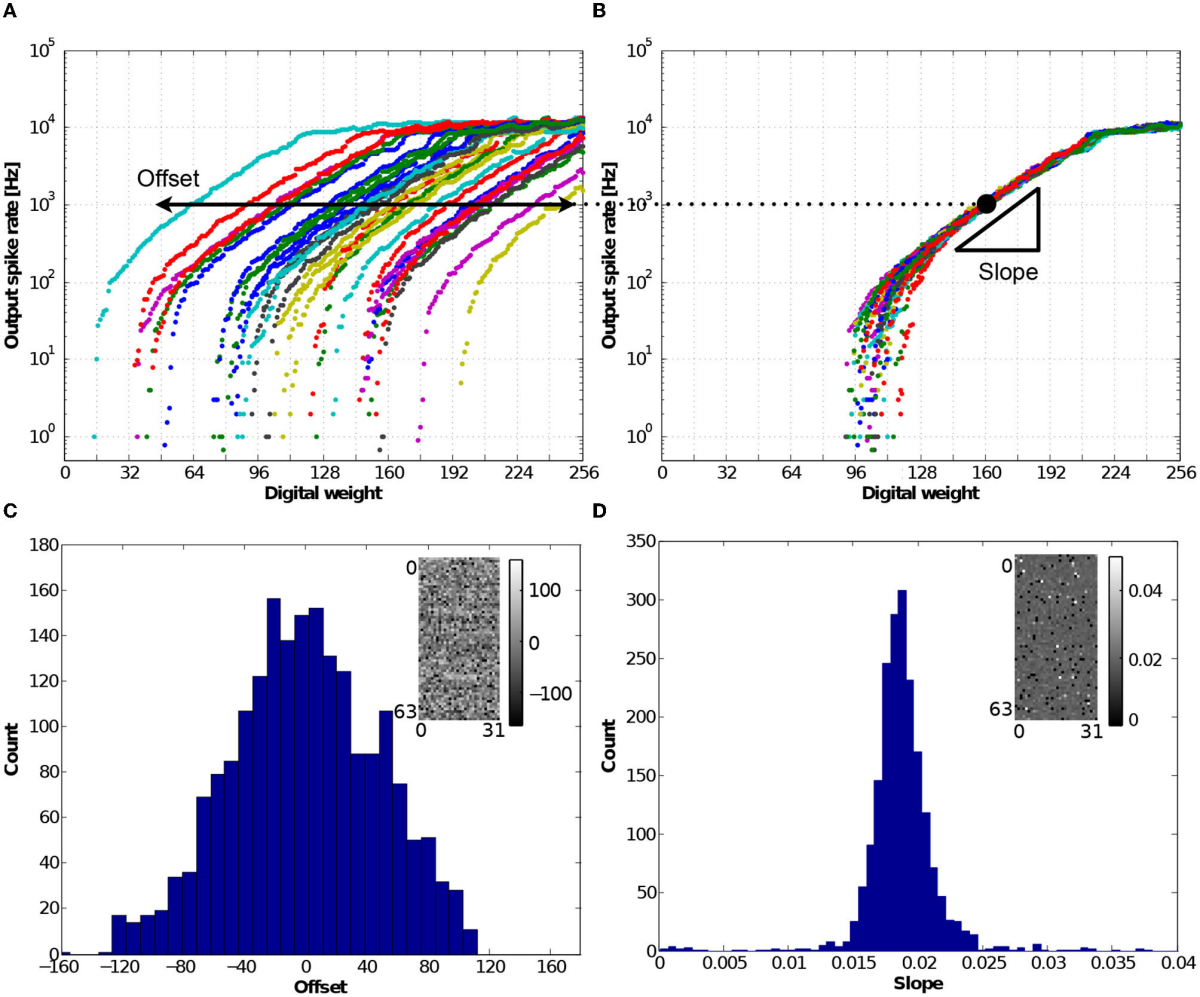
**(A)** Measured output frequency response curves as a function of eight-bit synaptic digital weight, which were measured from 32 neurons in the representative row. The input spike train was a 10,000 Hz mean-rate Poisson spike train in 1 s measurement. The result shows the offset of neuron activation caused by the threshold voltage mismatch of the transistor in the axon hillock circuit. **(B)** Offset compensated neuron responses aligned to the mean response. The slope is defined as the ratio of the output spike rate increments in a decade and the unit of digital weight. **(C)** Histogram of the offsets across a representative 2 k-neuron core. It shows the normal distribution with a wide variance across sample counts, while the inset shows a colormap representing the spatial distribution of offset; the brightest dot represents the most positive offset and the darkest dot represents the most negative offset. **(D)** Histogram of the slopes across the representative 2 k-neuron core. It has a normal distribution with a mean of 0.0185 and standard deviation of 0.0068, and its spatial distribution is drawn in the inset.

Analog-based neuron circuits designed with transistors in the subthreshold regime emulate biologically plausible neural systems efficiently with low power consumption, but they intrinsically exhibit large variations in neural responses owing to transistor mismatches. In the IFAT chip, one of the major sources of variation is the mismatch of the threshold voltage of a transistor in the axon hillock circuit. This mismatch results in a digital weight offset of the neural activation. Figure 13A shows the measured output spike rate responses from representative 32 neurons in the same row when the digital weights were varied from 0 to 255. Here, the input spike rate was 10,000 Hz, and the interspike intervals were distributed in the Poisson distribution. The offset is monitored as the digital weight at which the gain of the neural response is 0.1 (with an output spike rate of 10^3^). The digital weight offset can be compensated by synaptic weight learning in the address event domain (Park and Jung, 2020). Figure 13B shows the output spike rate responses when the weight offsets are compensated. The response curves are aligned to the mean of the 32 neural responses. The slope of the output spike rate increment over a decade to the digital weight shows the linearity of the synapse responses in the input-output transfer function in the linear response regime. For further analysis, we conducted measurements on a representative 2 k-neuron IFAT core, and the histograms of the offset and slope are shown in Figures 13C, D, respectively. The colormaps for 2 k-neurons (64 rows and 32 columns), drawn in the insets, represent the spatial distributions of the offset and slope in the array.

The calibration process shown above is effective to accommodate the relatively large variations in the subthreshold regime. However, it constitutes no hardware and software overhead at the inference. It is because the calibration is done offline, and the pre-distortion digital coefficients are stored externally, with the synapses dynamically instantiated (Park et al., 2017). In any case, the instantiation needs to be done as part of the HiAER-IFAT operation, and there is no cost for changing the digital entries in the lookup table based on the calibrated characteristics.

### 3.7. Linear synapse response model

The current injection into the leaky integrate-and-fire neuron model is formulated as follows:


(8) $$\begin{array}{l} I_{inj}=C_{mem}\frac{dV_{mem}}{dt}=g_{ext}\left(E_{ext}-V_{mem}\right)\\ +g_{inh}\left(E_{inh}-V_{mem}\right)\\ +g_{leak}\left(E_{L}-V_{mem}\right) \end{array}$$

where *C*_*mem*_ is the membrane capacitance, *V*_*mem*_ is the membrane voltage, *g*_*ext*_ and *g*_*inh*_ are the conductances of the excitatory and inhibitory synapses, *E*_*ext*_ and *E*_*inh*_ are the reversal potentials of the excitatory and inhibitory synapses, respectively, *g*_*leak*_ is the leak conductance, *E*_*L*_ is the leak voltage, and *V*_*mem*_ is the membrane voltage. Using a mean-rate approximation on a time scale of multiple action potentials, we can approximate the above terms to a simple linear neural response model, as follows:


(9) $$\begin{array}{l} I_{inj}=g_{ext}E_{ext}+g_{inh}E_{inh} \end{array}$$

With a first-order approximation, we assumed that the conductance is equal to the nominal synapse weight multiplied by the total number of spikes in the input spike trains:


(10) $$\begin{array}{l} g_{syn}\propto \underset{n}{\sum }f_{in,n}w_{n}=f_{in,eff}w_{nom} \end{array}$$

where *g*_*syn*_ is the conductance of the synapse, *f*_*in, n*_ is the frequency of the *n*_*th*_ input spike train, *w*_*n*_ is the synapse weight of the *n*_*th*_ input spike train, *f*_*in, eff*_ is the sum of all the input spike train frequencies, and *w*_*nom*_ is the nominal synapse weight. Given a first-order approximation, the output frequency is the sum of the excitatory and inhibitory synaptic input spike trains times the nominal synapse weight.


(11) $$\begin{array}{l} f_{out}={\left[G_{w_{nom}}f_{in,eff}w_{nom}\right]}^{+}={\left[G_{w_{nom}}\left(f_{ext,eff}w_{nom}-f_{inh,eff}w_{nom}\right)\right]}^{+} \end{array}$$

where *G*_*w*_*nom*__ denotes the gain-scaling factor at *w*_*nom*_. The gain-scaling factor, which is the frequency response gain, is defined as the ratio of the frequency response gain to the digital weight.

**Figure 14:**
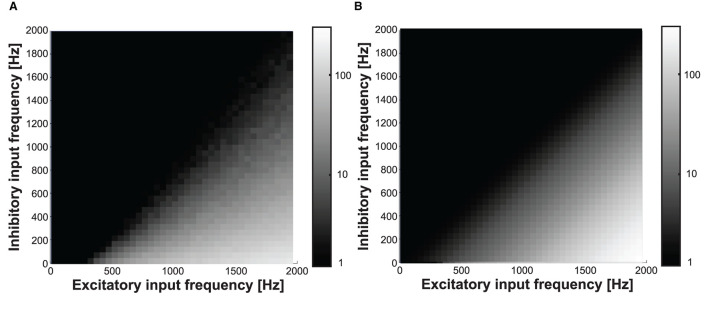
**(A)** Measured and **(B)** modeled output frequency while varying the excitatory and inhibitory input frequencies from 0 to 2,000 at digital weight of 80.

Figure 13 shows the measured (in Figure 14A) and modeled (in Figure 14B) output frequency response colormaps, while the excitatory and inhibitory synapse input frequencies are varied from 0 to 2,000 Hz at a nominal digital weight of 80. We used it as the model of the neuron response for the orientation tuning curve and boundary detection shown in the following sections.

### 3.8. Orientation tuning curve

**Figure 15:**
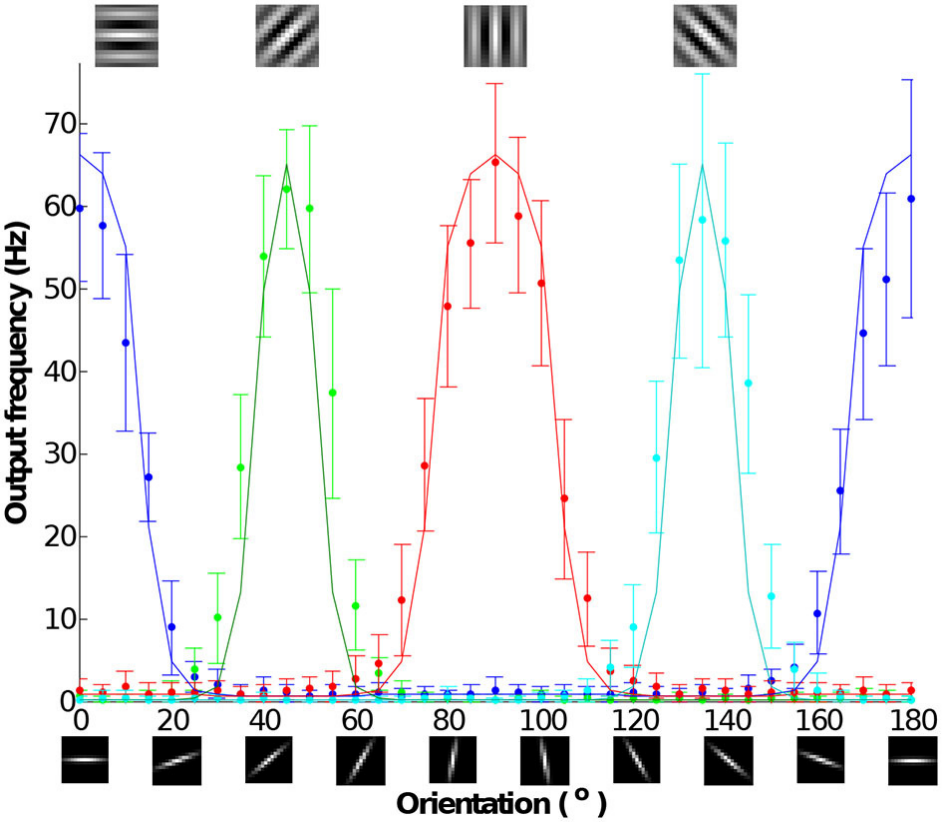
Measured tuning curves from the representative neuron with 15 × 15 pixel bar stimulus rotating orientation from 0 to 180° by 5° per each and four 15 × 15 pixel Gabor filters, each with 0, 45, 90, and 135°. Pixel intensity of the stimulus is translated as a synaptic input frequency ranging from 0 (darkest) to 63 (brightest). Pixel intensity of the filter is translated as a synapse weight. Each data point is the mean of 30 measurements each with 1 s stimulation. The solid lines show simulation models from the output frequency response model show in Figure 14.

An orientation tuning curve shows the firing rate selectivity of a neuron to stimuli with different orientations. It is a typical measurement used to characterize orientation selectivity in visual cortical neurons. Figure 15 shows the measured tuning curves of the IFAT chip. An output neural response is the measured result of the convolution of a stimulus and an orientated filter. Each data point is the mean of 30 measurements each with 1 s projection to a neuron. We used 15 × 15-pixel bar stimuli with rotations ranging from 0 to 180° in 5° steps. These stimuli were convolved into four Gabor patch orientations (0, 45, 90, and 135°). The pixel intensity of the stimuli is converted to input spike rates ranging from 0 (darkest) to 63 (brightest). The pixel intensity of a Gabor patch is translated into the synaptic strength of the input. Using Equation (11), the output frequency can be calculated as follows:


(12) $$\begin{array}{l} f_{out}={\left[\overset{15}{\underset{i=1}{\sum }}\overset{15}{\underset{i=1}{\sum }}f_{in_{i,j}}w_{i,j}\right]}^{+} \end{array}$$

where i and j are the indices of pixel positions, *f*_*out*_ is the output spike rate, *f*_*in*_ is the input synaptic spike rate, and *w* is the input synapse weight. Figure 15 shows that the simulation results drawn in solid lines lie within the range of the measured data points within one standard deviation.

### 3.9. Boundary detection

**Figure 16:**
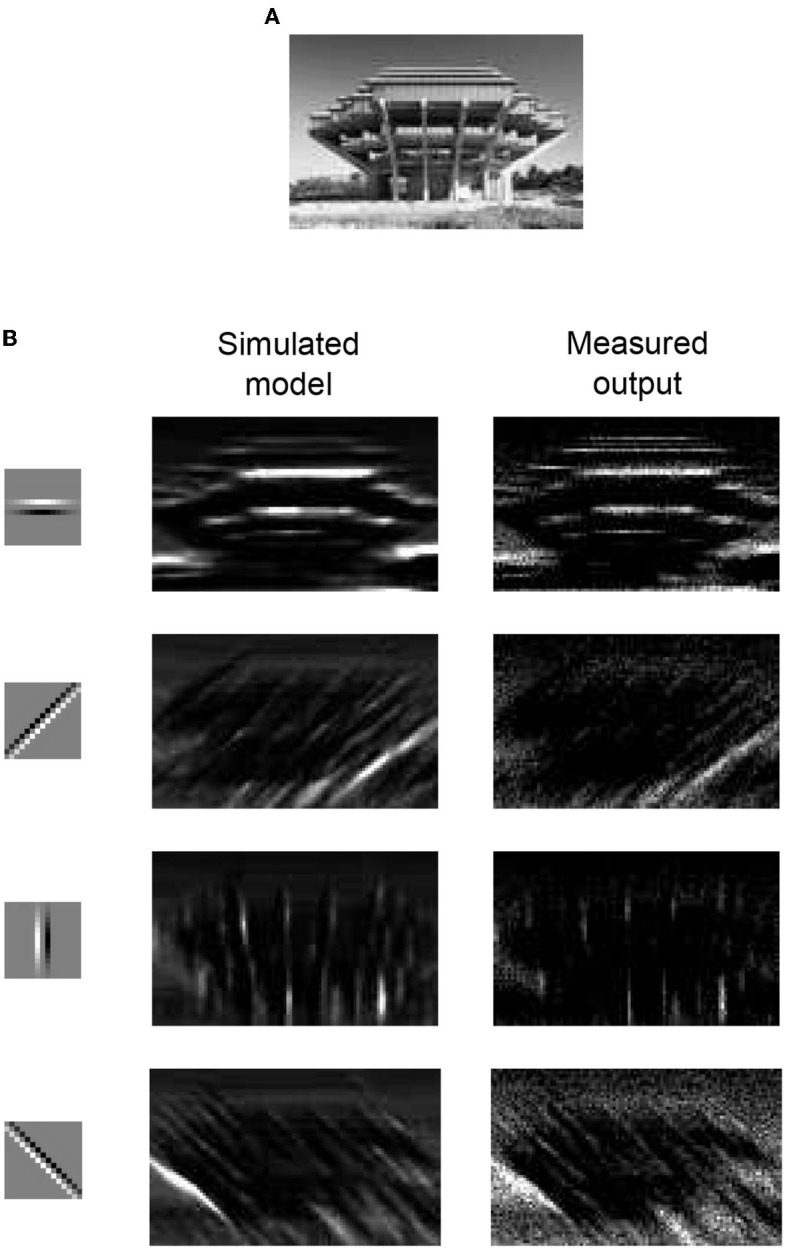
**(A)** Raw input image with a size of 113 × 75 pixels. **(B)** Boundary detection with simulated model and measurement results. The 15 × 15-pixel kernels used for the boundary detection are shown in the first column. For a simulation and measurement, a kernel presented at the same row is used. The simulation results and measured outputs are shown in the second and third columns, respectively.

Gabor-like local receptive fields are used to extract elementary visual features, such as oriented edges and corners, from images. This is an essential step for CNNs, which are a type of feed-forward neural network inspired by the biological multilayer perceptrons widely used in image recognition systems (Lecun et al., 1998). The layers in a CNN comprise feature maps and a subsequent spatial subsampling layer to down-sample raw image data. Here, we present an example of image boundary detection, which is an elementary component of a CNN. Image boundary detection was performed with an input image with a size of 113 × 75 pixels, as shown in Figure 16A. We used four edge detection kernels, each with a 15 × 15-pixel patch, as shown in Figure 16B in the first column. The experimental procedure was the same as that of the orientation tuning curve measurements. The stimulus was a 15 × 15 patch of a region in the image, and each pixel intensity of the patch was converted to an input synaptic event rate. The pixel intensity of an edge detection kernel is translated into synaptic weight. The convolution result of the image patch and an edge detection kernel were projected onto the representative neuron, and the output spike rate of the neuron was measured to reconstruct the filtered image output. Figure 16B in the second column shows the expected images, which were simulated using Equation (11). Figure 16B in the third column shows the measurement results for the IFAT neuron. The measurement results show that the reconstructed image from the measured output matches the expected images well. This shows that the IFAT neuron can be used as an essential unit for CNNs.

## 4. Discussion

Recently, many large-scale neuromorphic systems have been presented using various design approaches ranging from FPGAs and asynchronous digital to subthreshold analog design (Thakur et al., 2018). Such diverse approaches with their own design objectives make it difficult to compare large-scale neuromorphic systems quantitatively. We tried to compare neuromorphic processors, which are designed to extend to large-scale neuromorphic systems with a multi-chip routing architecture. Table 1 summarizes the measured characteristics of the IFAT chip in comparison with state-of-the-art works. It shows the IFAT chip has good area density and energy efficiency aspects.

**Table 1:** Comparisons with state-of-the-art works.

**References**	**Stromatias et al. (2013)**	**Merolla et al. (2014)**	**Davies et al. (2018)**	**Schemmel et al. (2010)**	**Yu et al. (2012b)**	**Benjamin et al. (2014)**	**Qiao et al. (2015)**	**This work**
Technology (*nm*)	130	28	14	180	130	180	28	90
Die size (*mm*^2^)	102	430	60	50	25	168	7.28^f^	16
Neuron type	Digital	Digital	Digital	Analog	Analog	Analog	Analog	Analog
Total number of neurons	~5,216^a^	1 M	130 k	512	65 k	65 k	1 k	65 k
Neuron area (μ*m*^2^)	N/A^a^	14.3^c^	400,000^d^	1,500	200	1,800	20	140
Throughput (Mevents/s)	5	N/A^b^	N/A	65	35	91	1,843^e^	73
Energy (J/Spike)	8 n	26 p	23.6 p	N/A	55 p	31.2 p	50 p^f^	22 p

a Software-instantiated leaky integrated and fire neuron.

b Internal connectivity.

c By multiplexing the neuron 256 times.

d When a core emulates 1,024 neural units.

e Simulation results.

f Reported in Thakur et al. (2018). N/A, Not Available.

The IFAT has been designed with an analog-based neuron and synapse circuit implemented with subthreshold conduction CMOS transistors. It achieved efficiency in power and area consumption with biologically plausible continuous analog temporal dynamics. However, the synaptic weight digitally encoded with an address event is stored in synaptic routing tables implemented in external memory, which is supported by HiAER-IFAT architecture (Park et al., 2017). It requires additional memory access to instantiate synaptic events, degrading energy efficiency. To address the issue, the synapse can be replaced with various emerging non-volatile memory devices such as ReRAM and magnetoresistive random access memory, which are recently presented for potential synaptic devices in analog neuromorphic hardware (Ielmini and Wong, 2018; Sun et al., 2018; Wang et al., 2018; Luo et al., 2020; Jang and Park, 2022; Tang et al., 2022; Wan et al., 2022). These emerging memory devices typically feature low-power and high-density compared to silicon-based CMOS logic circuits: a ReRAM device consumes about 0.1 pJ per switching operation (Ielmini and Wong, 2018). ReRAMs can be integrated with Silicon-based CMOS logic by using a monolithic 3D integration (Li et al., 2021). It means that synapses implemented by ReRAMs can be integrated on top of IFAT neurons and HiAER architecture, resulting in higher density and lower energy consumption.

## 5. Conclusion

In this paper, we presented a general-purpose neuromorphic processor that can serve as a basic computational building block for large-scale neuromorphic systems. The chip was fabricated using a 90-nm CMOS process and occupied a 4 × 4 *mm*^2^ die area. It is equipped with 65-k two-compartmental leaky integrate-and-fire neurons. Event-driven fully asynchronous circuits minimize the event communication latency, which is not bounded to any synchronized clock speed. In addition, the two-tier asynchronous micro-pipelining scheme maximizes the parallelization of event delivery to neurons in multiple rows; thus, resulting in a sustained throughput of 18.2 Mspikes/s per quadrant and 73 Mspikes/s for the chip. A high density of synapses and neurons was achieved by the single transistor synapse implementation and virtual synaptic wiring supported by the AER, resulting in 11.5 × 12.15 μ*m*^2^ integration for a neuron and four synapse types. An activity-driven asynchronous design enables the achievement of a system-level energy efficiency of 22 pJ per spike event. The proposed processor implemented biophysical details in compartmental conductance-based dynamics without compromising in area density and energy efficiency.

## Data availability statement

The original contributions presented in the study are included in the article/supplementary material, further inquiries can be directed to the corresponding author.

## Author contributions

JP and SH designed the chip and experiments, conducted the experiments, and wrote the paper. TY designed the chip and contributed to the discussions. EN contributed spike-based machine learning and inference tools and wrote the paper. GC designed the chip and experiments and wrote the paper. All authors contributed to the article and approved the submitted version.

## References

[B1] Akopyan F. Sawada J. Cassidy A. Alvarez-Icaza R. Arthur J. Merolla P.. (2015). TrueNorth: design and tool flow of a 65 mW 1 million neuron programmable neurosynaptic chip. IEEE Trans. Comput. Aided Des. Integr. Circuits Syst. 34, 1537–1557. 10.1109/TCAD.2015.2474396

[B2] Badoni D. Giulioni M. Dante V. Del Giudice P. (2006). “An aVLSI recurrent network of spiking neurons with reconfigurable and plastic synapses,” in IEEE International Symposium on Circuits and Systems, ISCAS 2006 (Kos), 1227–1230. 10.1109/ISCAS.2006.1692813

[B3] Benjamin B. Gao P. McQuinn E. Choudhary S. Chandrasekaran A. Bussat J.. (2014). Neurogrid: a mixed-analog-digital multichip system for large-scale neural simulations. Proc. IEEE 102, 699–716. 10.1109/JPROC.2014.2313565

[B4] Boahen K. A. (2000). Point-to-point connectivity between neuromorphic chips using address events. IEEE Trans. Circuits Syst. II 47, 416–434. 10.1109/82.842110

[B5] Brette R. Gerstner W. (2005). Adaptive exponential integrate-and-fire model as an effective description of neuronal activity. J. Neurophysiol. 94, 3637–3642. 10.1152/jn.00686.2005 16014787

[B6] Davies M. Srinivasa N. Lin T. H. Chinya G. Cao Y. Choday S. H.. (2018). Loihi: A neuromorphic manycore processor with on-chip learning. IEEE Micro 38, 82–99. 10.1109/MM.2018.112130359

[B7] Deiss S. R. Douglas R. J. Whatley A. M. (1999). A Pulse-Coded Communications Infrastructure for Neuromorphic Systems. MIT Press, 157–178.

[B8] Destexhe A. Mainen Z. F. Sejnowski T. J. (1998). “Kinetic models of synaptic transmission,” in Methods in Neuronal Modelling, From Ions to Networks (MIT Press), 1–25.

[B9] Detorakis G. Sheik S. Augustine C. Paul S. Pedroni B. U. Dutt N.. (2018). Neural and synaptic array transceiver: a brain-inspired computing framework for embedded learning. Front. Neurosci. 12:583. 10.3389/fnins.2018.00583 30210274 PMC6123384

[B10] Fragnière E. Schaik A. V. Vittoz E. (1997). Reactive components for pseudo-resistive networks. Elect. Lett. 33, 19131914. 10.1049/el:19971348

[B11] Frenkel C. Legat J.-D. Bol D. (2019). MorphIC: a 65-nm 738k-synapse/mm^2^ quad-core binary-weight digital neuromorphic processor with stochastic spike-driven online learning. IEEE Trans. Biomed. Circuits Syst. 13, 999–1010. 10.1109/TBCAS.2019.2928793 31329562

[B12] Furber S. Lester D. Plana L. Garside J. Painkras E. Temple S.. (2012). Overview of the SpiNNaker system architecture. IEEE Trans. Comput. 62, 2454–2467. 10.1109/TC.2012.14236188479

[B13] Fusi S. Mattia M. (1999). Collective behavior of networks with linear (VLSI) integrate-and-fire neurons. Neural Comput. 11, 633–652. 10.1162/089976699300016601 10085424

[B14] Goldberg D. H. Cauwenberghs G. Andreou A. G. (2001). Probabilistic synaptic weighting in a reconfigurable network of VLSI integrate-and-fire neurons. Neural Netw. 14, 781–793. 10.1016/S0893-6080(01)00057-0 11665770

[B15] Groschner L. N. Malis J. G. Zuidinga B. Borst A. (2022). A biophysical account of multiplication by a single neuron. Nature 603, 119–123. 10.1038/s41586-022-04428-3 35197635 PMC8891015

[B16] Hodgkin A. L. Huxley A. F. (1952). A quantitative description of membrane current and its application to conduction and excitation in nerve. J. Physiol. 117:500. 10.1113/jphysiol.1952.sp004764 12991237 PMC1392413

[B17] Ielmini D. Wong H.-S. P. (2018). In-memory computing with resistive switching devices. Nat. Electron. 1, 333–343. 10.1038/s41928-018-0092-231997981

[B18] Imam N. Akopyan F. Arthur J. Merolla P. Manohar R. Modha D. S. (2012). “A digital neurosynaptic core using event-driven QDI circuits,” in 18th IEEE International Symposium on Asynchronous Circuits and Systems (ASYNC) (Lyngby), *2012*, 25–32. 10.1109/ASYNC.2012.12

[B19] Imam N. Cleland T. A. (2020). Rapid online learning and robust recall in a neuromorphic olfactory circuit. Nat. Mach. Intell. 2, 181–191. 10.1038/s42256-020-0159-4 PMC11034913 38650843

[B20] Indiveri G. Chicca E. Douglas R. (2006). A VLSI array of low-power spiking neurons and bistable synapses with spike-timing dependent plasticity. IEEE Trans. Neural Netw. 17, 211–221. 10.1109/TNN.2005.860850 16526488

[B21] Indiveri G. Linares-Barranco B. Hamilton T. Schaik A. V. Etienne-Cummings R. Delbruck T.. (2011). Neuromorphic silicon neuron circuits. Front. Neurosci. 5:73. 10.3389/fnins.2011.00073 21747754 PMC3130465

[B22] Izhikevich E. M. (2003). Simple model of spiking neurons. IEEE Trans. Neural Netw. 14, 1569–1572. 10.1109/TNN.2003.820440 18244602

[B23] Jang Y. Park J. (2022). Area and energy efficient joint 2T SOT-MRAM-based on diffusion region sharing with adjacent cells. IEEE Trans. Circuits Syst. 69, 1622–1626. 10.1109/TCSII.2021.3126638

[B24] Koch C. (1999). Biophysics of Computation: Information Processing in Single Neurons. Computational neuroscience. New York, NY: Oxford University Press.

[B25] Lazzaro J. Wawrzynek J. Mahowald M. Sivilotti M. Gillespie D. (1993). Silicon auditory processors as computer peripherals. IEEE Trans. Neural Netw. 4, 523–528. 10.1109/72.217193 18267754

[B26] Lecun Y. Bottou L. Bengio Y. Haffner P. (1998). Gradient-based learning applied to document recognition. Proc. IEEE 86, 2278–2324. 10.1109/5.726791

[B27] Li Y. Tang J. Gao B. Yao J. Xi Y. Li Y.. (2021). “Monolithic 3D integration of logic, memory and computing-in-memory for one-shot learning,” in 2021 IEEE International Electron Devices Meeting (IEDM) (San Francisco, CA), 21.5.1–21.5.4. 10.1109/IEDM19574.2021.9720534

[B28] Lin J. Merolla P. Arthur J. Boahen K. (2006). “Programmable connections in neuromorphic grids,” in 49th IEEE International Midwest Symposium on Circuits and Systems, MWSCAS 2006, Vol. 1 (San Juan, PR), 80–84.

[B29] London M. Häusser M. (2005). Dendritic computation. Annu. Rev. Neurosci. 28, 503–532. 10.1146/annurev.neuro.28.061604.135703 16033324

[B30] Luo T. Wang X. Qu C. Lee M. K. F. Tang W. T. Wong W.-F.. (2020). An FPGA-based hardware emulator for neuromorphic chip with RRAM. IEEE Trans. Comput. Aided Des. Integr. Circuits Syst. 39, 438–450. 10.1109/TCAD.2018.2889670

[B31] Mahowald M. (1994). An Analog VLSI System for Stereoscopic Vision, Vol. 265. Springer. 10.1007/978-1-4615-2724-4

[B32] Martin A. Nystrom M. (2006). Asynchronous techniques for system-on-chip design. Proc. IEEE 94, 1089–1120. 10.1109/JPROC.2006.87578916041988

[B33] Martin A. J. Nystrom M. Papadantonakis K. Penzes P. I. Prakash P. Wong C. G.. (2003). “The lutonium: a sub-nanojoule asynchronous 8051 microcontroller,” in Proceedings Ninth International Symposium on Asynchronous Circuits and Systems, 2003 (Vancouver, BC), 14–23. 10.1109/ASYNC.2003.1199162

[B34] Mead C. (1990). Neuromorphic electronic systems. Proc. IEEE 78, 1629–1636. 10.1109/5.58356

[B35] Merolla P. Arthur J. Akopyan F. Imam N. Manohar R. Modha D. S. (2011). “A digital neurosynaptic core using embedded crossbar memory with 45pJ per spike in 45nm,” in IEEE Custom Integrated Circuits Conference (CICC) (San Jose, CA), *2011*, 1–4. 10.1109/CICC.2011.6055294

[B36] Merolla P. A. Arthur J. V. Alvarez-Icaza R. Cassidy A. S. Sawada J. Akopyan F.. (2014). A million spiking-neuron integrated circuit with a scalable communication network and interface. Science 345, 668–673. 10.1126/science.1254642 25104385

[B37] Mihalas S. Niebur E. (2009). A generalized linear integrate-and-fire neural model produces diverse spiking behaviors. Neural Comput. 21, 704–718. 10.1162/neco.2008.12-07-680 18928368 PMC2954058

[B38] Millner S. Grübl A. Meier K. Schemmel J. Schwartz M.-O. (2011). “A VLSI implementation of the adaptive exponential integrate-and-fire neuron model,” in Advances in Neural Information Processing Systems, NIPS 2010, Vol. 23 (Vancouver, BC: MIT Press), 1642–1650.

[B39] Mitchell S. J. Silver R. (2003). Shunting inhibition modulates neuronal gain during synaptic excitation. Neuron 38, 433–445. 10.1016/S0896-6273(03)00200-9 12741990

[B40] Muller D. E. Bartky W. S. (1957). A Theory of Asynchronous Circuits I. University of Illinois, Graduate College, Digital Computer Laboratory.

[B41] Nair V. Hinton G. E. (2010). “Rectified linear units improve restricted Boltzmann machines,” in Proceedings of the 27th International Conference on Machine Learning (ICML-10) (Haifa: Omnipress), 807–814.

[B42] Neckar A. Fok S. Benjamin B. V. Stewart T. C. Oza N. N. Voelker A. R.. (2019). Braindrop: a mixed-signal neuromorphic architecture with a dynamical systems-based programming model. Proc. IEEE 107, 144–164. 10.1109/JPROC.2018.2881432

[B43] Nelson M. E. (1994). A mechanism for neuronal gain control by descending pathways. Neural Comput. 6, 242–254. 10.1162/neco.1994.6.2.242

[B44] Painkras E. Plana L. A. Garside J. Temple S. Galluppi F. Patterson C.. (2013). SpiNNaker: a 1-W 18-core system-on-chip for massively-parallel neural network simulation. IEEE J. Solid-State Circuits 48, 1943–1953. 10.1109/JSSC.2013.2259038

[B45] Park J. Ha S. Yu T. Neftci E. Cauwenberghs G. (2014). “65k-neuron 73-Mevents/s 22-pJ/event asynchronous micro-pipelined integrate-and-fire array transceiver,” in IEEE Biomedical Circuits and Systems Conference (BioCAS) (Lausanne), 675–678. 10.1109/BioCAS.2014.6981816

[B46] Park J. Jung S.-D. (2020). Presynaptic spike-driven spike timing-dependent plasticity with address event representation for large-scale neuromorphic systems. IEEE Trans. Circuits Syst. I 67, 1936–1947. 10.1109/TCSI.2020.2966884

[B47] Park J. Yu T. Joshi S. Maier C. Cauwenberghs G. (2017). Hierarchical address event routing for reconfigurable large-scale neuromorphic systems. IEEE Trans. Neural Netw. Learn. Syst. 28, 2408–2422. 10.1109/TNNLS.2016.2572164 27483491

[B48] Qiao N. Mostafa H. Corradi F. Osswald M. Stefanini F. Sumislawska D.. (2015). A reconfigurable on-line learning spiking neuromorphic processor comprising 256 neurons and 128k synapses. Front. Neurosci. 9:141. 10.3389/fnins.2015.00141 25972778 PMC4413675

[B49] Ramakrishnan S. Wunderlich R. Hasler J. George S. (2013). Neuron array with plastic synapses and programmable dendrites. IEEE Trans. Biomed. Circuits Syst (Hsinchu), 7, 631–642. 10.1109/TBCAS.2013.2282616 24144669

[B50] Ramakrishnan S. Wunderlich R. Hasler P. (2012). “Neuron array with plastic synapses and programmable dendrites,” in IEEE Biomedical Circuits and Systems Conference (BioCAS), 2012, 400–403. 10.1109/BioCAS.2012.6418412 24144669

[B51] Schemmel J. Bruderle D. Grubl A. Hock M. Meier K. Millner S. (2010). “A wafer-scale neuromorphic hardware system for large-scale neural modeling,” in Proceedings of 2010 IEEE International Symposium on Circuits and Systems (ISCAS) (Paris), 1947–1950. 10.1109/ISCAS.2010.5536970

[B52] Schmitt S. Klähn J. Bellec G. Grübl A. Güttler M. Hartel A.. (2017). “Neuromorphic hardware in the loop: training a deep spiking network on the brainscales wafer-scale system,” in 2017 International Joint Conference on Neural Networks (IJCNN) (Anchorage, AK), 2227–2234. 10.1109/IJCNN.2017.7966125

[B53] Sharp T. Galluppi F. Rast A. Furber S. (2012). Power-efficient simulation of detailed cortical microcircuits on SpiNNaker. J. Neurosci. Methods 210, 110–118. 10.1016/j.jneumeth.2012.03.001 22465805

[B54] Silver R. Boahen K. Grillner S. Kopell N. Olsen K. L. (2007). Neurotech for neuroscience: unifying concepts, organizing principles, and emerging tools. J. Neurosci. 27, 11807–11819. 10.1523/JNEUROSCI.3575-07.2007 17978017 PMC3275424

[B55] Sivilotti M. A. (1991). Wiring considerations in analog VLSI systems, with application to field-programmable networks (Ph.D. thesis), California Institute of Technology, Pasadena, CA, United States.

[B56] Stromatias E. Galluppi F. Patterson C. Furber S. (2013). “Power analysis of large-scale, real-time neural networks on SpiNNaker,” in The 2013 International Joint Conference on Neural Networks (IJCNN) (Dallas, TX), 1–8. 10.1109/IJCNN.2013.6706927

[B57] Sun Z. Ambrosi E. Bricalli A. Ielmini D. (2018). Logic computing with stateful neural networks of resistive switches. Adv. Mater. 30:1802554. 10.1002/adma.201802554 30079525

[B58] Tang Q. He Z. Liu F. Wang Z. Zhou Y. Zhang Y.. (2022). “HAWIS: Hardware-Aware automated WIdth Search for accurate, energy-efficient and robust binary neural network on ReRAM dot-product engine,” in 2022 27th Asia and South Pacific Design Automation Conference (ASP-DAC) (Taipei), 226–231. 10.1109/ASP-DAC52403.2022.9712542

[B59] Thakur C. S. Molin J. L. Cauwenberghs G. Indiveri G. Kumar K. Qiao N.. (2018). Large-scale neuromorphic spiking array processors: a quest to mimic the brain. Front. Neurosci. 12:891. 10.3389/fnins.2018.00891 30559644 PMC6287454

[B60] Vogelstein R. J. Mallik U. Vogelstein J. T. Cauwenberghs G. (2007). Dynamically reconfigurable silicon array of spiking neurons with conductance-based synapses. IEEE Trans. Neural Netw. 18, 253–265. 10.1109/TNN.2006.883007 17278476

[B61] Wan W. Kubendran R. Schaefer C. Eryilmaz S. B. Zhang W. Wu D.. (2022). A compute-in-memory chip based on resistive random-access memory. Nature 608, 504–512. 10.1038/s41586-022-04992-8 35978128 PMC9385482

[B62] Wang Y. Liu S.-C. (2013). Active processing of spatio-temporal input patterns in silicon dendrites. IEEE Trans. Biomed. Circuits Syst. 7, 307–318. 10.1109/TBCAS.2012.2199487 23853330

[B63] Wang Z. Joshi S. Savel'ev S. Song W. Midya R. Li Y.. (2018). Fully memristive neural networks for pattern classification with unsupervised learning. Nat. Electron. 1, 137–145. 10.1038/s41928-018-0023-2

[B64] Yang S. Gao T. Wang J. Deng B. Lansdell B. Linares-Barranco B. (2021). Efficient spike-driven learning with dendritic event-based processing. Front. Neurosci. 15:601109. 10.3389/fnins.2021.601109 33679295 PMC7933681

[B65] Yu T. Cauwenberghs G. (2010). “Log-domain time-multiplexed realization of dynamical conductance-based synapses,” in Proceedings of 2010 IEEE International Symposium on Circuits and Systems (ISCAS) (Paris), 2558–2561. 10.1109/ISCAS.2010.5537114

[B66] Yu T. Park J. Joshi S. Maier C. Cauwenberghs G. (2012a). “65k-neuron integrate-and-fire array transceiver with address-event reconfigurable synaptic routing,” in IEEE Biomedical Circuits and Systems Conference (BioCAS) (Hsinchu), *2012*, 21–24. 10.1109/BioCAS.2012.6418479

[B67] Yu T. Park J. Joshi S. Maier C. Cauwenberghs G. (2012b). “Event-driven neural integration and synchronicity in analog VLSI,” in Annual International Conference of the IEEE Engineering in Medicine and Biology Society (EMBC) (San Diego, CA), *2012*, 775–778. 10.1109/EMBC.2012.6346046 23366007

